# MEFV mutations - therapeutic guides or red herrings?

**DOI:** 10.1186/1546-0096-13-S1-P129

**Published:** 2015-09-28

**Authors:** K Warrier, L Cliffe, L McDermott, S Rangaraj

**Affiliations:** 1 Nottingham University Hospitals NHS Trust, Paediatric Rheumatology, Nottingham, UK; 2 Nottingham University Hospitals, Child Health, Nottingham, UK; 3 Nottingham University Hospitals NHS Trust, Immunology, Nottingham, UK

## Background/question

Familial Mediterranean Fever (FMF) is a hereditary inflammatory disorder characterised by self-resolving attacks of fever and serositis common in populations from Mediterranean ancestry[1]. Mutations affecting MEFV gene is believed to be responsible for the disease phenotype[1]. The correlation between the genotype and phenotype is not very strong, indicating the presence of other modifying factors which alter clinical manifestation. We describe 2 children with autoinflammtory symptoms, who had MEFV mutations, the significance of which we are unsure of.

## Methods

A 13-year-old Polish girl of Romani descent of non-consanguineous parents presented with one-week history of fever, chest pain and vomiting. She was alert; but pale, tachycardic and hypotensive with normal capillary filling time and warm peripheries, despite saline boluses and broad spectrum antibiotics. She had no rash, mouth ulcers, lymphadenopathy or hepatosplenomegaly. Her investigations showed leucocytosis (22.2 x 10^9^/L) with neutrophilia (20.2), hypoalbuminaemia (19g/L) and high inflammatory markers (CRP 222mg/L/ ESR 142mm/hour). She had past history of 3 -4 similar episodes every year since 5 years of age, one of which needed a lengthy admission to the Intensive Care Unit. She was then advised a 3-day course of oral Prednisolone at the onset of each episode, to which she did not respond this time. She continued to spike temperature in excess of 39°C daily for the next five weeks. Her inflammatory markers went up further, along with high ferritin (3729 microg/L) and LDH (1003 U/L) with persistent hypoalbuminaemia, leucocytosis with neutrophilia and thrombocytosis (platelets 597 x 10^9^/L) and worsening anaemia (Haemoglobin 63 g/L). Investigations for an underlying immunodeficiency, infection, autoimmune disorder, macrophage activation and inflammatory bowel disease were negative. Her serum Amyloid A (SAA) was 798 mg/L. A diagnosis of an unclassified autoinflammatory disorder was made by exclusion, for which she was commenced on Anakinra at 100mg daily. Her symptoms settled and investigations normalised dramatically, including SAA. Her autoinflammtory genetics screen later revealed she is positive for MEFV E148Q mutation. Because her symptoms were not typical of FMF and were well controlled on Anakinra, treatment was not altered.

A 2-year-old boy of non-consanguineous parents of Indian origin was referred with recurrent episodes of high fevers lasting 4 days, since nine months of age with frequency increasing from 8 weeks to every 3 weeks. A typical episode starts with him rubbing ears and nose, followed by fever up to 40°c, with little response to Paracetamol and Ibuprofen. He had no other symptoms during or between these episodes. Two maternal nieces had recurrent fevers when young, which improved with tonsillectomy. His examination was normal with no rashes, mouth ulcers, lymphadenopathy or hepatosplenomegaly, although he had large tonsils with small cervical lymph nodes. He had raised inflammatory markers during the episodes. With a possible diagnosis of PFAPA syndrome, he was advised a single dose of Prednisolone at 2mg per kg at the start of an attack. He responded to Prednisolone during subsequent attacks, but parents were concerned about worsening frequency. His autoinflammtory genetics screen revealed a mutation in E148Q in exon 2 of the MEFV gene. His clinical features were not typical of FMF without any evidence of serositis and good response to Prednisolone, which prompted us to withhold Colchicine for the time being.

## Results and conclusion

Although E148Q is one of the one of the five most frequent MEFV mutations in the classically affected ethnic groups, it is considered to be the mildest and least penetrant with no reported incidence of amyloidosis[1]. Some studies[3] report a high frequency of E148Q mutation (21%) in Indian population used as control, an ancestry both these patients may well share. Although it may cause FMF when associated with certain other MEFV mutations, homozygosity for E148Q alone must be insufficient to produce FMF[3]. That leaves us with the question of the significance of the mutations in these children, who do not have classical symptoms of FMF but are significantly symptomatic, especially with regards to switching therapy when there is a response to the existing agent.

## Consent to publish

Written informated consent for publication of their clinical details was obtained from the patient/parent/guardian/relative of the patient.
